# Effects of Low and High FODMAP Diets on Human Gastrointestinal Microbiota Composition in Adults with Intestinal Diseases: A Systematic Review

**DOI:** 10.3390/microorganisms8111638

**Published:** 2020-10-23

**Authors:** Doris Vandeputte, Marie Joossens

**Affiliations:** 1 Center for Microbiology, VIB, 3000 Leuven, Belgium; 2 Department of Microbiology, Immunology and Transplantation, Rega Institute, KU Leuven—University of Leuven, 3000 Leuven, Belgium; 3 Meinig School of Biomedical Engineering, Cornell University, Ithaca, NY 14853, USA; 4 Department of Biochemistry and microbiology (WE10), Laboratory of Microbiology, Ghent University, 9000 Ghent, Belgium; marie.joossens@ugent.be

**Keywords:** irritable bowel syndrome, Crohn’s disease, ulcerative colitis, gut bacteria, bifidogenic effect, dysbiosis, FODMAP, oligosaccharides, fermentation, prebiotics

## Abstract

A diet high in non-digestible carbohydrates is known to promote health, in part through its effect on the gut microbiome. While substantially proven for healthy individuals, these effects are more ambiguous in subjects with intestinal diseases. At the same time, a diet low in these fermentable carbohydrates, the low FODMAP (acronym for Fermentable Oligo-, Di-, Mono-saccharides, And Polyols) diet, is gaining popularity as a treatment option for symptom relief in irritable bowel syndrome and inflammatory bowel disease. There are, however, several indications that this diet induces effects opposite to those of prebiotic supplementation, resulting in gut microbiome changes that might be detrimental. Here, we provide a systematic review of the effects of low and high FODMAP diets on human gastrointestinal microbiota composition in adults with intestinal diseases, through literature screening using the databases PubMed, Embase, and Web of Science. We summarize study findings on dietary impact in patients, including the effect on bacterial taxa and diversity. In general, similar to healthy subjects, restricting non-digestible carbohydrate intake in patients with intestinal diseases has opposite effects compared to prebiotic supplementation, causing a reduction in bifidobacteria and an increase in bacteria associated with dysbiosis. Future studies should focus on assessing whether the induced microbial changes persist over time and have adverse effects on long-term colonic health.

## 1. Introduction

According to the prebiotic concept, non-digestible carbohydrate consumption can have health benefits through effects on the intestinal microbiota. Although stimulating health-promoting bacteria originally has received the most attention, other mechanisms such as reducing unfavorable bacteria or functions should be considered as well [1,2]. Studies in healthy individuals have repeatedly shown that non-digestible carbohydrate consumption results in the increase of members of the Bifidobacteriaceae and Lactobacillaceae families. These bacteria are able to either ferment the prebiotic components themselves (in the case of primary degraders) or stimulate the growth of other species by metabolizing the prebiotic and thereby providing substrates via cross-feeding. They are thought to promote health through various mechanisms, including the production of short-chain fatty acids [1,3,4]. Recently, several other effects of prebiotic consumption have been described as well, including the stimulation of favorable or reduction of unfavorable bacteria often associated with a healthy microbiome, such as *Faecalibacterium prausnitzii* and *Akkermansia muciniphila* [3,4,5,6]. It is, however, unclear if the same effects apply in dysbiotic microbiomes of diseased individuals.

Fermentation of non-digestible carbohydrates at the small intestine and colon can, moreover, cause side-effects that overlap with symptoms characteristic for chronic intestinal conditions like irritable bowel syndrome (IBS) and inflammatory bowel disease (IBD) [7,8,9]. Water retention and gas production through bacterial fermentation are the main mechanisms by which these components induce or worsen symptoms like bloating and diarrhea [7,8,9]. Increased intestinal permeability has also been suggested to play a role [7]. Inspired by observed symptom reduction in IBS patients, a diet low in fermentable carbohydrates became a support strategy for several intestinal diseases [10,11]. This dietary strategy, known as the low FODMAP diet because of its reduction in fermentable oligo-, di-, monosaccharides, and polyols [7], clashes with the prebiotic concept and related findings. In addition, some studies even indicate that the low FODMAP diet leads to opposite compositional effects in patients with intestinal disorders, namely a reduction of health-associated bacteria, such as bifidobacteria, and shifts similar to those noted in dysbiotic systems [12,13]. Given the important role of the gut microbiota for intestinal and systemic health, these results suggest possible detrimental effects on long-term colonic health.

A comprehensive overview of the available literature is, however, missing, making it hard to assess the consistency of these findings. This knowledge gap hampers further research and impedes treatment options in clinical practice. Here, we provide a systematic review of all studies investigating the effects of fermentable carbohydrates on gut microbiota composition in intestinal diseases. This review summarizes the results of intervention studies applying diets either low or high in fermentable oligo-, di-, mono-saccharides, and polyols (abbreviated as ‘FODMAPS’) in adults, as assessed through enumeration of bacterial taxa in fecal samples. With this overview, we provide guidelines for further study and assessment of the gut microbiome with the low FODMAP diet and associated health consequences.

## 2. Materials and Methods

### 2.1. Literature Search

A systematic search of the literature was conducted using the databases PubMed, Embase, and Web of Science up until 10 April 2020. Eligible studies included those reporting the effects of diets low or high in FODMAP ingredients on human gastrointestinal microbiota composition in adults with intestinal diseases.

Inclusion based on the criterion of a low or high FODMAP diet was evaluated as either (i) the use of the term FODMAP to describe the applied diet by the authors, or (ii) the increased/decreased consumption of the dietary compounds described as oligosaccharides, fructans, fructo-oligosaccharides, oligo-fructose, inulin, galactans, galacto-oligosaccharides, disaccharides, lactose, monosaccharides, fructose, polyols, sorbitol, xylitol, mannitol, maltitol, and isomalt. Included studies needed to report results on individuals with colonic diseases, described as inflammatory bowel disease, colitis, enteritis, inflammatory intestinal disease, inflammatory enteropathy, bowel inflammation, intestinal inflammation, ileitis, colonitis, Crohn’s disease, ulcerative colitis, diverticular disease, diverticulitis, diverticulosis, colon cancer, colorectal cancer, colonic polyps, irritable bowel syndrome, irritable colon, spastic colon, nervous colon, mucous colitis, or spastic bowel.

We excluded (i) studies only reporting results based on children, (ii) studies only reporting results based on animal (non-human) experimentation or obtained through in vitro methods (such as cell lines or fermentation), (iii) studies without data on gut microbiome composition, (iv) studies using synbiotics (as the prebiotic effect could not be disentangled from the effect of the probiotic), or those only describing responder/non-responder analyses, (v) reviews, editorials, commentaries, or studies only reported in abstract form, (vi) studies reported in languages other than English.

A search strategy for PubMed, Embase, and Web of Science was developed based on the specified inclusion criteria and the structure of the respective databases. In order to include all relevant literature and decrease the changes of missing relevant trials, we adopted a broad screening approach. The final search strings were as follows: PubMed: (((“Diet, Carbohydrate-Restricted”[Mesh] OR Carbohydrate-Restricted-Diet*[tiab] OR Low-Carbohydrate-Diet*[tiab] OR Carbohydrate-Restricted-High-Protein-Diet*[tiab] OR Low-Carbohydrate-High-Protein-Diet*[tiab] OR (“Dietary Carbohydrates”[Mesh:NoExp] AND “Diet Therapy”[Mesh:NoExp]))) AND “Colonic Diseases”[Mesh]) AND (“Gastrointestinal Microbiome”[Mesh] OR “Gastrointestinal Microbiome”[tiab]); Embase: (‘low fodmap diet’ OR ‘low carbohydrate diet’ OR oligosaccharide OR fructan OR inulin OR galactan OR disaccharide OR lactose OR monosaccharide OR fructose OR polyol OR sorbitol OR xylitol OR mannitol OR maltitol OR isomalt) AND (‘colon disease’/exp OR ‘irritable colon’/exp) AND ‘intestine flora’/exp NOT (‘animal experiment’ OR mouse OR rat OR rodent) NOT (‘conference abstract’:it OR review:it) AND english:la; Web of Science Core Collection: TOPIC: (‘low FODMAP diet’ OR ‘low carbohydrate diet’ OR oligosaccharide OR fructan OR inulin OR galactan OR disaccharide OR lactose OR monosaccharide OR fructose OR polyol OR sorbitol OR xylitol OR mannitol OR maltitol OR isomalt) AND TOPIC: (Colonic disease OR colon disease OR inflammatory bowel disease OR colitis OR enteritis OR inflammatory intestinal disease OR inflammatory enteropathy OR bowel inflammation OR intestinal inflammation OR ileitis OR colonitis OR Crohn’s disease OR ulcerative colitis OR diverticular disease OR diverticulitis OR diverticulosis OR colon cancer OR colorectal cancer OR colonic polyps OR irritable bowel syndrome OR irritable colon OR spastic colon OR nervous colon OR mucous colitis OR spastic bowel) AND TOPIC: ((gastrointestinal OR intestinal OR intestine OR gut OR abdominal OR bowel OR duodenum OR colon) AND (microbiota OR flora OR microbes OR microbiome)) AND DOCUMENT TYPES: (Article OR Letter) NOT TITLE: (animal experiment OR mouse OR mice OR rat OR rodent OR piglet OR chicken) NOT TITLE: (Obesity OR ‘in vitro’) AND LANGUAGE: (English) NOT DOCUMENT TYPES: (Book Chapter OR Book Review OR Data Paper OR Proceedings Paper OR Review), subsequently refined by: [excluding] Web of Science categories: (obstetrics gynecology or marine freshwater biology or cardiac cardiovascular systems or agriculture multidisciplinary or biochemical research methods or dentistry oral surgery medicine or pediatrics or energy fuels or agriculture dairy animal science or engineering biomedical or veterinary sciences or parasitology or plant sciences or materials science multidisciplinary or mathematical computational biology or medicine legal or radiology nuclear medicine medical imaging or reproductive biology or neurosciences or zoology or fisheries or allergy or dermatology); Timespan: 1995–2020.

DV and MJ independently screened the obtained records for inclusion into the final review based on the predefined criteria. Inconsistencies were resolved by discussion.

### 2.2. Data Extraction

DV and MJ extracted data from the included papers. Extracted data included the following: study type, sample size (per protocol analysis), duration of intervention, intestinal disease, diet type, microbiome data type, effect on bacterial taxa, effect on bacterial diversity, effect on bacterial density/load, effect on symptoms, and microbiome data availability.

Microbiome data discussion was limited to findings obtained with fecal samples.

## 3. Results

**Figure 1:**
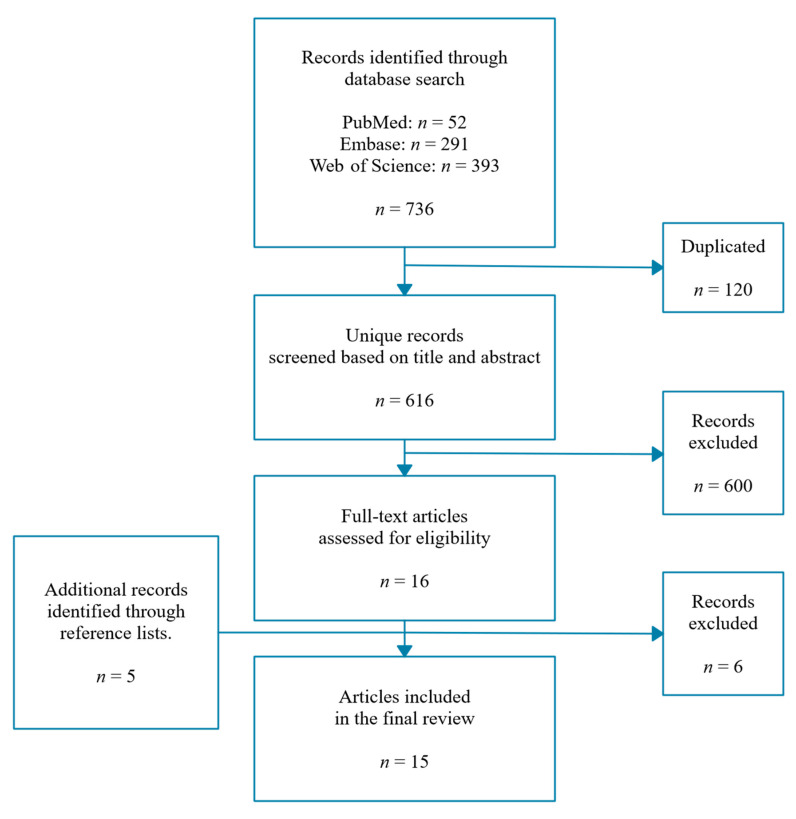
Flow diagram showing the results of the systematic literature search.

**Figure 2:**
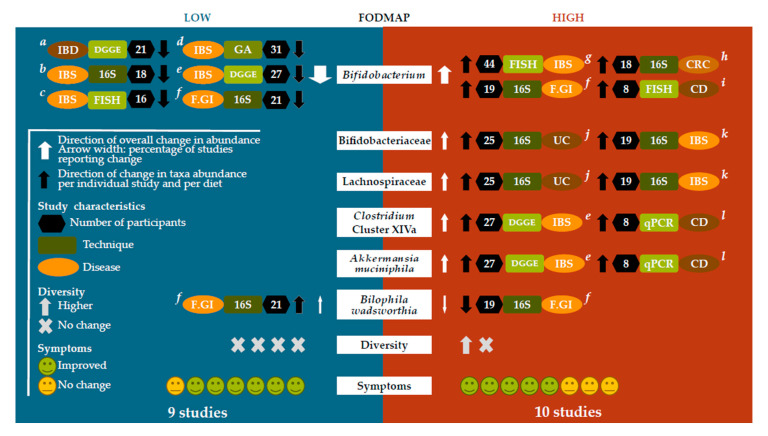
Graphical summary of the main findings. Overview of significant findings with low FODMAP diets (left, 9 studies) versus high fermentable carbohydrate intake (right, 10 studies) for taxonomic groups reported at two instances (*Bifidobacterium*, Bifidobacteriaceae, Lachnospiraceae, *Clostridium* cluster XIVa, *Akkermansia muciniphila* and *Bilophila wadsworthia*), as well as diversity measures (Diversity) and symptom severity (Symptoms). Studies not included in the figure did not report any significant change regarding the respective parameter. All individual study results are referenced (white letters: a. Cox et al., *Gastroenterology*, 2020; b. Staudacher et al., *Gastroenterology*, 2017; c. Staudacher et al., *Journal of Nutrition*, 2012; d. Bennet et al., *Gut*, 2018; e. Halmos et al., *Gut*, 2015; f. Huaman et al., *Gastroenterology*, 2018; g. Silk et al., *Alimentary Pharmacology & Therapeutics*, 2009; h. Xie et al., *Nutrition*, 2019; i. Lindsay et al., *Gut*, 2006; j. Valcheva et al., *Gut Microbes*, 2019; k. McIntosh et al., *Gut*, 2017; l. Halmos et al., *Clinical and Translational Gastroenterology*, 2016). Abbreviations: IBD: inflammatory bowel disease; IBS: irritable bowel syndrome; F.GI: functional gastro-intestinal disorder; UC: ulcerative colitis; CRC: colorectal cancer; CD: Crohn’s disease; DGGE: denaturing gradient gel electrophoresis; GA: GA-map™ Dysbiosis test; 16S: 16S ribosomal amplicon profiling; FISH: fluorescent in situ hybridization; qPCR: quantitative polymerase chain reaction.

A systematic search of the literature was conducted using the databases PubMed, Embase, and Web of Science. Eligible studies included those reporting effects of diets low or high in FODMAP ingredients on human intestinal microbiota composition in adults with intestinal diseases. A total of 15 studies [14,15,16,17,18,19,20,21,22,23,24,25,26,27,28] were withheld on this screening basis, some of which included diets of varying non-digestible carbohydrate content (Figure 1). These included 13 randomized controlled trials, and 2 open-label studies, with one of the latter assessing multiple doses. Most prevailing diseases were irritable bowel syndrome (seven studies) and inflammatory bowel diseases (six studies; focusing on Crohn’s disease (CD) patients only (*n* = 4), CD and ulcerative colitis (UC) patients combined (*n* = 1), and UC patients only (*n* = 1)), with varying levels of disease activity (Table S1). The amount of studies discussing high or low FODMAP diets was almost equal (10 and 9 studies, respectively). Studies investigating the low FODMAP diet often did so using patients with irritable bowel syndrome, while diets high in FODMAP content were frequently used for patients with inflammatory bowel diseases or colorectal cancer. From all 15 studies, we extracted results about changes in gut microbiome composition upon prebiotic supplementation or non-digestible carbohydrate restriction. After giving an overview of all study results, we zoom in on taxa reported at least twice, each time discussing supplementation trial results followed by restriction trial results (see the graphical summary in Figure 2).

The described microbial changes upon prebiotic supplementation in subjects with intestinal diseases are largely similar to those reported for healthy individuals [29]. A bifidogenic effect is noted repeatedly (6/10 studies). Sporadically, increased Ruminococcaceae (2/10 studies), Lachnospiraceae (2/10 studies), *Clostridium* cluster XIVa (2/10 studies), and, *Akkermansia muciniphila* (2/10 studies) as well as decreased *Bilophila wadsworthia* (1/10 studies) are found. In contrast to studies in healthy individuals [6,30,31,32,33,34], increases in *Faecalibacterium prausnitzii* were not detected in any of the included studies. Restriction of non-digestible carbohydrate consumption generally had opposite effects. It resulted in decreased *Bifidobacterium* or Bifidobacteriaceae abundance (6/9 studies), decreased Lactobacillaceae (1/9 studies), Propionibacteriaceae (1/9 studies), *Clostridium* cluster IV (1/9 studies), *Faecalibacterium prausnitzii* (1/9 studies), as well as increased *Bilophila wadsworthia* (1/9 studies), Clostridiales family XIII incertae sedis (1/9 studies), and *Porphyromonas IV* (1/9 studies).

Augmented bifidobacterial abundance is a well-documented effect of prebiotic supplementation in healthy and diseased individuals [29,35,36]. Bifidobacteria are known as primary degraders of polysaccharides and are thus provided with a selective advantage when such dietary components are abundant [36]. The consequences of fermentable carbohydrate restriction on bifidobacteria are, however, less clear [24]. Together, the studies considered here, seem to indicate that carbohydrate restriction indeed leads, as expected, to Bifidobacterium reduction in diseased individuals. Benjamin et al. even found an inverse correlation between Bifidobacterium abundance and carbohydrate intake [14]. Within prebiotic studies, the size of the so-called bifidogenic effect is often found to depend on the baseline Bifidobacterium abundance [37,38]. Staudacher et al. demonstrated that the reverse is true when prebiotic carbohydrates are restricted in IBS patients: people with higher fecal bifidobacteria at baseline had a greater reduction upon the low FODMAP diet [24]. Multiple studies in healthy volunteers as well as patients with intestinal diseases link Bifidobacterium presence and abundance to general and intestinal health. Bifidobacterium has been shown to limit pathogenic colonization [39,40] and to have immunomodulatory properties [41,42]. In addition, lower Bifidobacterium abundance has been linked to abdominal pain in healthy individuals [43] and IBS patients [44]. Given that most of the studies indicated improvement of symptoms (mostly in functional disorders), these results hold an interesting paradox, specifically, that a dietary intervention that seems to benefit the patient regarding symptoms also results in a reduction in bifidobacteria, which are positively correlated with pain relief. An interesting question is therefore whether probiotic supplementation in addition to fermentable carbohydrate restriction could enhance symptom response.

FODMAP consumption, within the normal diet or through supplementation, is further known to stimulate species from the Lactobacillaceae and Lachnospiraceae families, as well as *Clostridium* clusters, through direct fermentation or cross-feeding in healthy individuals [45,46]. Although a consistent signal is absent, similar effects are noted here in a diseased setting, with increased Ruminococcaceae (2/10 studies), Lachnospiraceae (2/10 studies), and *Clostridium* cluster XIVa (2/10 studies) with high FODMAP diets. There is some sporadic evidence for the reduction of these bacterial groups with fermentable carbohydrate restriction as well: 1/9 studies report decreased Lactobacillaceae, Propionibacteriaceae, and *Clostridium* cluster IV amounts. Noteworthy is the absence of a consistent signal for the cross-feeding butyrate-producer and proposed health-indicator *F. prausnitzii* [47,48]. Only one study reports a decrease of this species with a low FODMAP diet in IBS patients. It might be that this highly prevalent (>90% of individuals) and abundant (generally >5%) species is not notably affected because of disease-associated factors. Levels of *F. prausnitzii* have been found to be decreased in all diseases included here, namely irritable bowel syndrome, inflammatory bowel disease, and colorectal cancer, as well as in other situations deviating from normal gastro-intestinal health, such as obesity, celiac disease, and frailty in elderly [48]. Therefore, inflammation or other disease-associated factors might play a more important role and reduce or overrule dietary effects.

We here find some evidence for increased abundance of *Akkermansia muciniphila* after prebiotic supplementation in diseased subjects (2/10 studies), similar to observations in animal studies [5]. The reverse effect, namely decreased abundance with carbohydrate restriction, was not noted in any of the included studies. *Akkermansia muciniphila* is a mucus-degrading bacterium of the phylum Verrucomicrobia. Although its main energy source is host-associated mucus, it might be indirectly stimulated by dietary FODMAP components though syntrophic interactions (e.g., acetate degradation) [5]. Precise mechanisms of prebiotic stimulation are, however, unclear. Like *F. prausnitzii*, *Akkermansia* is considered a health indicator species [49]. Reduced numbers of this bacterium have been reported in IBS, IBD, and CRC patients, as well as other diseases, such as, obesity and type 2 diabetes [5,49]. Consequently, also here, disease-associated factors might partly cancel or obscure dietary effects.

Lastly, an inverse association of *Bilophila wadsworthia* abundance with fermentable carbohydrate consumption comes forward from the study results. This is also in line with reports in healthy individuals [50,51,52]. *Bilophila wadsworthia*, a member of the Proteobacteria phylum, uses sulfate as the main electron-acceptor during anaerobic respiration [53,54,55] and seems to thrive on a diet high in animal-derived protein and fat [51,56,57,58]. *B. wadsworthia* is often classified as a pathobiont, due to its frequent detection in patient groups [59,60,61], its ample virulence potential [62,63], and the potentially deleterious nature of its metabolic end-products [57,64]. Several mechanisms are proposed to underlie the observed reduction in *Bilophila* abundance with FODMAP components, namely, (i) the induction of quantitative or qualitative changes in bile acid production away from sulfite-containing taurine-conjugated components [50,65], (ii) a suppressive effect on hydrogen sulfide production [66,67], or simply (iii) the acidic environment created by carbohydrate fermentation [50,64].

Next to taxonomical changes, we extracted information about microbial diversity and total bacterial load. High fiber diets are generally associated with higher species diversity in healthy individuals [68,69,70]. This is, however, likely a result of long-term dietary habits [70,71,72]. Here, only 2/10 and 4/9 studies applying a high or low FODMAP diet, respectively, assessed species diversity. Diversity increased in one out of two studies assessing a high FODMAP diet, while no changes were noted in all other occasions. Since FODMAPs are a major energy source for colonic microbes, bacterial loads can be expected to vary with FODMAP intake. Such effects are indeed noted in healthy individuals, where high fiber diets or prebiotic supplementation are associated with higher bacterial densities and/or higher fecal volumes [73,74,75,76,77], and low FODMAP diets are associated with reduced bacterial densities (but not necessarily colonic volumes) compared to habitual or comparator diets [22,52]. Here, only Halmos et al. and Staudacher et al. reported data on total bacterial densities, measured as the number of copies of the 16S rRNA gene by qPCR per gram of feces, in Crohn’s disease and IBS patients, respectively. They did not observe any difference in total bacterial densities between a low FODMAP diet and their respective control diets. Of note, reduced bacterial densities might not only stem from less bacterial growth as a consequence of reduced substrate availability, but could also be due to dilution of fecal material, a point worth considering, especially with the bulking capacities of some prebiotics and in circumstances of intestinal disease.

Although not the focus of this review, we extracted information on symptom improvement. Of 10 studies assessing a high FODMAP diet, 6 reported symptom improvements, 3 reported no significant change regarding symptoms, and 1 did not assess this parameter. Most low FODMAP trials also registered positive outcomes regarding symptoms, with six studies noting improvements versus one reporting no change and two unassessed.

## 4. Discussion

In general, restricting the intake of fermentable carbohydrates has the opposite effect of prebiotic supplementation in patients with intestinal disease, with a reduction in bifidobacteria and shifts similar to those observed with dysbiosis. Species diversity and bacterial density were not affected in most studies considering these parameters, but data are very limited.

It is not known whether the impact of the low FODMAP diet on the gut microbiota is long-term, as longitudinal follow-up is only seldomly included in current studies. In addition, most of the studies only report microbiota changes at the end of a restrictive period (4–8 weeks) but do not consider the suggested reintroduction phase, in which fermentable carbohydrate restriction becomes less stringent [78]. With the exception of Harvie et al. [25], who found persistent symptom improvement in IBS patients with the reintroduction of FODMAPs, yet did not observe any microbial changes upon the FODMAP diet or 3 months thereafter (possibly due to a loss of samples after a technical issue), the long-term impact of a low FODMAP diet is currently unassessed. It is thought that microbiota are resilient to change in the absence of environmental stressors [56,79,80,81], which would suggest the restoration of the initial microbiome once a normal diet is resumed. However, several studies also indicate the loss of microbial species and functions with the reduction of fiber intake [82], suggesting that such changes might be irreversible. Further research is necessary to illuminate whether these short-term changes persist over time.

Strengths of this review are the inclusion of both low and high FODMAP diets, considering the full spectrum of carbohydrate intake and strengthening the conclusions through the opposing observations; the focus on intestinal diseases, limiting the evaluation to the target population; and the limitation to an adult microbiome, which—in contrast to that of children—is considered to be stable over time. By including only microbiome data from patients that completed a study per protocol, we furthermore provide the first clear view of the impact on the dysbiotic microbiome using all available patient data before and after actual treatment.

A major limitation of the current review is the small amount of studies included, as well as the limited sample size of some of these studies. Interpersonal variation of the human gut microbiome is large and often exceeds the effect of a dietary intervention. Some of the studies discussed here did not include a sufficient number of samples to detect signals beyond individual variation. Occasionally, a too small sample size resulted in the avoidance of multiple testing correction, therefore resulting in the reporting of significant effects for taxa previously reported to differ between groups. This might inflate the importance of the detected differences. The mixed, and sometimes even conflicting, results are probably—at least partially—due to the different study protocols and to the heterogeneity of the fermentable carbohydrate content in the applied prebiotic, low FODMAP, and comparator diets, as well as differences in sample collection, storage, and analysis methodology. Another weakness is that this review only considered taxonomical information at genus level or higher. Information on species or strain level, or functional assessments, would be useful but is not yet available due to the limited use of shotgun metagenomic sequencing. In addition, the included studies only sporadically measured bacterial densities, but none considered fecal mass, hampering the assessment of total bacterial load. Despite its availability, metabolomic data was also not considered yet could be informative, as it might reveal a more pronounced signal, given that McIntosh et al. found greater separation between a low FODMAP and a high FODMAP diet in the metabolome than the microbiome. Unfortunately, a meta-analysis of the study results was not possible due to several factors. First, none of the studies made the microbiome data publicly available. Second, processing differed largely among studies, complicating a meta-analysis substantially.

A few recommendations can be made based on this work. Ideally, future studies investigating this subject should run a power analysis based on the previously reported effect sizes to include sufficient individuals to detect the foreseen effects. Enumeration of the microbiome should be done using the latest techniques, including shotgun metagenomics, to allow functional analysis at a finer taxonomical level [83], and/or absolute quantitation techniques, to allow a transition from relative to absolute abundance estimation with associated advantages for comparative analysis and the detection of species–species or metadata–species associations [84,85,86,87,88,89,90,91]. While there are several methods to perform absolute abundance profiling, those based on measures of bacterial density could additionally provide an estimation of total bacterial load when combined with fecal mass/volume measurements and in this way reveal differences in the total productive capacity of the investigated microbial ecosystems. If resources are limited and microbiome results are only secondary, bacteria known to differ between patients and healthy controls or different diets could be targeted specifically by qPCR. Metabolomics might complement microbiome analysis and provide further mechanistical insights [92,93]. To compare findings and allow meta-analyses, it is important that samples are collected, stored, processed, and analyzed in a consistent manner, using appropriate computational biology tools. Standardization efforts conducted by large microbiome consortia could guide study protocols [94,95,96]. In addition, authors should deposit their raw data in public repositories to ensure future data availability. Both authors and editors bear a responsibility to ensure data sharing in order to aid scientific progress in the long run [97].

Next to these methodological and practical considerations, the results of this review suggest several future research directions. While a low FODMAP diet generally leads to a reduction of the health-associated *Bifidobacterium*, and a more dysbiotic microbiome composition, it is still unclear whether this effect persists over time or has any detrimental effects on long-term colonic health. Although far from straightforward, as this requires the adoption of a longitudinal study design as well as long-term, extensive sampling, future studies should focus on answering these questions.

Given the potential adverse effect on health, the microbial changes induced by a low FODMAP diet should raise some concern. Supplementation with probiotics could be considered to partly counteract these changes. Staudacher et al. have successfully applied such a strategy, leading to increased bifidobacteria numbers while maintaining symptom improvements [26]. Alternatively, a reverse strategy of increasing non-digestible carbohydrate intake could be applied. A few studies have investigated the hypothesis that prebiotic administration initially activates the fermentative metabolism of colonic microbiota, increasing gas production, and that this early effect is later followed by an adaptation of the microbiota with a reduction in net gas production [17,98,99]. Trials with galacto-oligosaccharides showed frequency and volume of evacuated gas increased during the first 3 weeks of prebiotic supplementation, only to decrease to baseline levels afterwards. An increase in the relative abundance of butyrate producers was noted, which correlated inversely with the volume of evacuated gas [98]. An adaptation in microbiota metabolism toward low gas producing pathways was shown in a subsequent study [99]. Huaman et al. compared both approaches, a low FODMAP diet versus galacto-oligosaccharide supplementation, and showed symptom improvements for both treatments in a four-week random controlled trial (RCT). Of note, symptoms reappeared immediately after quitting the low FODMAP diet, while patients of the prebiotic arm experienced reduced symptoms for 2 additional weeks [17]. This suggests prebiotic supplementation might be a viable treatment option for those who can endure the initial start-up phase. An important point to consider is that both dietary strategies show individually variable responses [10,100,101,102]. Personal factors, including the gut microbiome, which is highly variable between individuals [103,104,105], likely determine treatment outcome. Microbiome screening might aid clinicians in selecting the most promising option. Valeur et al. [106] and Chumpitazi et al. [107] have provided first insights into the microbiome characteristics associated with treatment success of a low FODMAP diet in adults and children with IBS, respectively, yet more research is needed to introduce this into clinical practice. To the best of our knowledge, similar responder analyses have not been carried out for prebiotics.

## Supplementary Materials

The following are available online at https://www.mdpi.com/2076-2607/8/11/1638/s1, Table S1. Overview of studies and microbiome results.

## Abbreviations

CD	Crohn’s Disease
CRC	Colorectal Cancer
DGGE	Denaturing Gradient Gel Electrophoresis
FISH	Fluorescence In Situ Hybridization
FODMAP	Fermentable Oligo-, Di-, Monosaccharides, and Polyols
Funct. GI	Functional Intestinal Disorders
IBD	Inflammatory Bowel Disease
IBS	Irritable Bowel Syndrome
OL	Open Label
PP	Per protocol
qPCR	quantitative Polymerase Chain Reaction
RCT	Randomized Controlled Trial
